# The Prevalence of Diabetes Mellitus Type II (DMII) in the Multiple Sclerosis Population: A Systematic Review and Meta-Analysis

**DOI:** 10.3390/jcm12154948

**Published:** 2023-07-27

**Authors:** Vasileios Giannopapas, Lina Palaiodimou, Dimitrios Kitsos, Georgia Papagiannopoulou, Konstantina Stavrogianni, Athanasios Chasiotis, Maria Kosmidou, John S. Tzartos, George P. Paraskevas, Daphne Bakalidou, Georgios Tsivgoulis, Sotirios Giannopoulos

**Affiliations:** 1 Second Department of Neurology, Attikon University Hospital, School of Medicine, National & Kapodistrian University of Athens, 15784 Athens, Greece; bgiannopapas@gmail.com (V.G.); lina_palaiodimou@yahoo.gr (L.P.); georgiapap22@hotmail.com (G.P.); stavrogianni.k@gmail.com (K.S.); thanosch1@gmail.com (A.C.); jtzartos@gmail.com (J.S.T.); geoprskvs44@gmail.com (G.P.P.); tsivgoulisgiorg@yahoo.gr (G.T.); 2 Physical Therapy Department, University of West Attica, 12210 Athens, Greece; dafbak@otenet.gr; 3 Laboratory of Neuromuscular and Cardiovascular Study of Motion (Lanecasm), 12243 Athens, Greece; 4 Department of Physiology, Faculty of Medicine, University of Ioannina, 45110 Ioannina, Greece; 5 Department of Internal Medicine, Faculty of Medicine, University of Ioannina, 45110 Ioannina, Greece; mkosmid@uoi.gr

**Keywords:** multiple sclerosis, diabetes mellitus II, autoimmune disorders, vascular comorbidity

## Abstract

**Introduction**: The interactions between Diabetes Mellitus type II (DMII) and Multiple Sclerosis (MS) lead to higher levels of fatigue, higher risk of physical disability, faster cognitive decline, and in general a lower quality of life and a higher frequency of depression compared to the general population. All of the above accelerate the disability progression of patients with MS, reduce the patients’ functional capacity, and further increase their psychological and economic burden. **Methods**: This systematic review and meta-analysis aims to calculate the prevalence of DMII in the MS population. Following PRISMA guidelines, a thorough search of the Medline Pubmed, Cochrane Library, and Scopus databases was performed, focusing on the frequency of DMII in the MS population. **Results**: A total of 19 studies were included in the synthesis. The results of the main meta-analysis of random effects using R studio 3.3.0 for Windows and the Meta r package showed that the prevalence of DMII in the MS population is 5% (95% CI [0.03, 0.07], 19 studies, I^2^ = 95%, p_Q_ < 0.001). Additional subgroup analysis based on region showed a difference of 4.4% (I^2^ = 95.2%, p_Q_ < 0.001), p_subgroupdifference_ = 0.003) between European and non-European participants, while demographic- and MS-specific characteristic (EDSS, Disease Duration) did not seem to affect the prevalence of DMII in the MS population (*p* = 0.30, *p* = 0.539, *p* = 0.19, *p* = 0.838). No publication bias was discovered (Egger’s *p* test value: 0.896). **Conclusions**: Even though the prevalence of DMII in the MS population is lower than 10% (the reported prevalence of DMII in the general population) the interactions between the two conditions create significant challenges for MS patients, their caregivers, and physicians. DΜΙΙ should be systematically recorded in the case of MS patients to clearly delineate any potential relationship between the two conditions. Additionally, more structured studies investigating the interactions of MS and DMΙΙ as well as the direction of the causation between those two conditions are necessary in order to gain a deeper insight into the nature of the interaction between MS and DMII.

## 1. Introduction

Multiple sclerosis (MS) is the most common neuroinflammatory and neurodegenerative demyelinating disease, affecting approximately 2.8 million people worldwide. A significant percentage of the MS population presents with one or more comorbid conditions (e.g., depression, anxiety, systematic erythymatoide lupus, and other). Autoimmune, cerebrovascular, and cardiovascular comorbidities in the MS population are being studied, mainly on their interaction with the disease’s neuroinflammatory and neurodegenerative progression and the patient’s disability status. Apart from that, in many cases comorbid conditions may lead to accelerated disease progression and higher levels of fatigue, as well as increased pharmacological, psychological, and economic burden [1,2].

In the general population, the overall prevalence of Diabetes Mellitus type II (DMII) is approximately 537 million adults worldwide [3]. There are several studies examining possible underlying the pathological mechanisms of DMII that may lead to the emergence of MS, while a respective percentage of MS patients develop DMII due to unknown, as of today, etiology. In relation with MS symptomatology, patients with DMII appear to have higher levels of fatigue, higher risk of physical disability, faster cognitive decline, are more likely to adopt a sedentary lifestyle, have a higher body mass index and a higher prevalence of depressive symptoms compared to the general population, and, in general, a lower quality of life. All of the above may accelerate the disability progression of patients with MS (PwMS), reduce their functional capacity, and further increase their psychological and economic burden [4,5,6,7].

The aim of this systematic review and meta-analysis is to calculate the prevalence of DMII in the MS population and the possible relationship of MSspecific characteristics (i.e., EDSS, disease duration). The rationale behind the decision to examine the prevalence is the negative interaction of the two conditions, mainly on the MS patients’ functionality and cognitive status. Because this is a meta-analysis of proportions, randomized controlled trials and observational studies including consecutive PwMS that either examined the prevalence of DMII or presented the percentage of DMII participants were included. 

## 2. Methods 

### 2.1. Study Design, Search Strategy, and Selection Criteria

A systematic literature review was conducted to identify all published research regarding DMII in MS patients by two independent authors (V.G., L.P.). The PICO search included P: Multiple sclerosis patients I: not applicable, C: not applicable, O: cases of diabetes mellitus type II. Records were retrieved from three separate databases (Medline, Scopus, and Cochrane Library) with no filter application regarding date and language of publication, or record type. The complete search algorithm is provided in the Supplementary Material. Related records and reference lists were screened for any potentially relevant studies. All disagreements were resolved after discussion with the corresponding author (S.G.).

Identified records were screened by two independent authors (V.G., L.P.) based on prespecified inclusion/exclusion criteria. The inclusion criteria included (i) Definite MS diagnosis (Mc Donald’s criteria 2018) and (ii) proportion (percentage or count) of DMII cases. Excluded from further analysis were (i) studies with unspecified type of diabetes, (ii) case series, (iii) case reports, and (iv) studies with purposive sampling. An aggregated meta-analysis was performed, including randomized controlled trials, observational studies, and cohort studies reporting the proportion of DMII cases in PwMS. The results of the meta-analysis are presented in accordance with the Preferred Reporting Items for Systematic Reviews and Meta-analyses [8]. The pre-specified study protocol has been registered in the International Prospective Register of Ongoing Systematic Reviews, PROSPERO (CCRD42023421012).

### 2.2. Quality Control and Bias Assessment

Eligible studies underwent quality control and bias assessment with the use of the Johanna Briggs Institute checklist for cross-sectional studies and the PLOS ONE for observational studies [9,10]. The quality control and bias assessment was performed by two independent authors (V.G., L.P.) and any disagreements were resolved via consensus and discussion with the corresponding author (S.G.).

### 2.3. Outcomes

The predefined outcome measure was the proportion of DMII cases in PwMS. In addition, potential differences in demographic characteristics (age, region) and MS related characteristics (EDSS, Disease Duration) among PwMS and comorbid DMII were evaluated.

### 2.4. Statistical Analysis

The prevalence of DMII in PwMS was generated using the metaprop function (Meta package version 6.5-0) [11] for R using the Maximum likelihood method and the pseudo-logistic function (plogit) because the recorded proportions were between 2% and 7%. Heterogeneity between included studies was assessed with the Cochran Q (sig. level 0.1) and I^2^ statistics [12]. The random effects model was employed for the meta-analysis. Publication bias across individual studies was assessed in cases where more than four studies were included in each analysis, using funnel plot inspection and the Egger’s linear regression test [13]. All statistical analyses were carried out using RStudio for Windows version 3.3.0 [14].

## 3. Results

### 3.1. Literature Search and Included Studies

**Figure 1:**
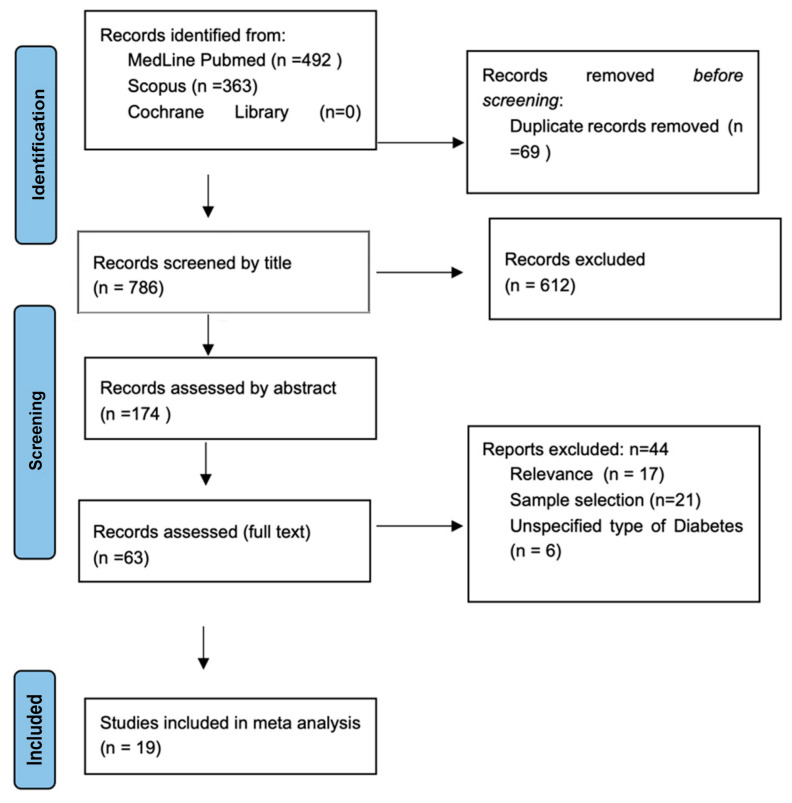
Prisma Flow-chart.

The systematic search produced a total of 855 results from both Medline and Scopus databases, while the search on the Cochrane Library returned 0 results (Figure 1). After excluding duplicates and out of scope articles, 63 records were considered eligible for inclusion and were assessed in full. Finally, 19 articles [15,16,17,18,19,20,21,22,23,24,25,26,27,28,29,30,31,32,33] were identified, including a total of 15,360 PwMS (Table 1).

### 3.2. Quality Control of Included Studies

Eligible studies underwent quality control and bias assessment with the use of the Johanna Briggs Institute checklist for cross-sectional studies and the PLOS ONE for observational studies. A total score of 89% was recorded from the PLOS ONE checklist and 93% from the JBI checklist, which are indicative of high quality (ST1, ST2).

### 3.3. Overall and Subgroup Analyses

**Figure 2:**
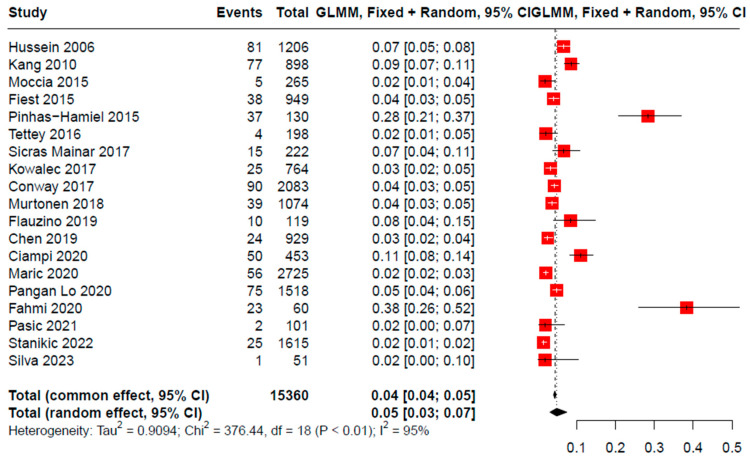
Pooled prevalence of DMII cases among PwMS [15,16,17,18,19,20,21,22,23,24,25,26,27,28,29,30,31,32,33].

**Figure 3:**
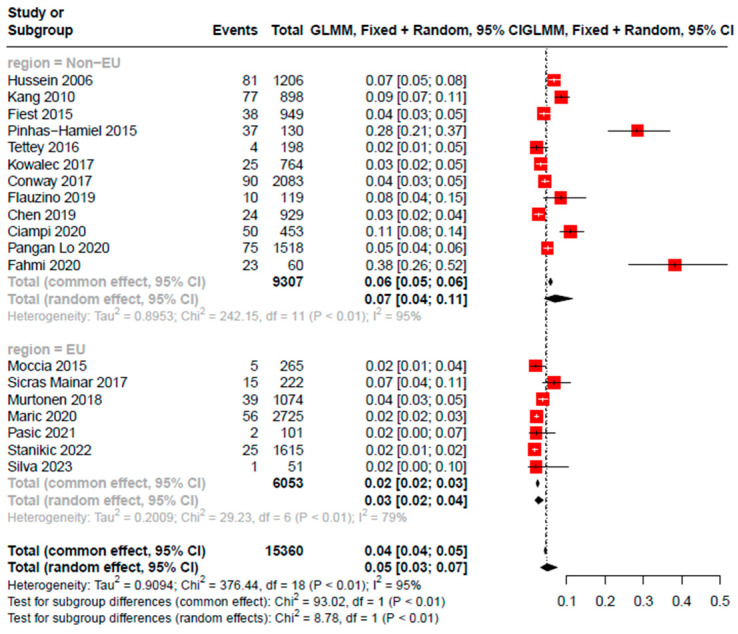
Subgroup analysis based on region [15,16,17,18,19,20,21,22,23,24,25,26,27,28,29,30,31,32,33].

A total of 15,360 PwMS were included in the meta-analysis. The mean age of the participants in the included studies was 46.00 years and the frequency of female gender was 71.18% participants. Regarding MS-specific characteristics, EDSS had a minimum of 1 and a maximum of 5.5, while disease duration had a minimum value of 3 years and a maximum value of 43.1 years The pooled prevalence rate of DΜΙΙ was found to be 5% (95% CI [0.03, 0.07], 19 studies, I^2^ = 95%, p_Q_ < 0.001) (Figure 2). Additionally, a subgroup analysis was performed based on region. The included studies were divided into two distinct groups, European region and non-European region. Statistically significant differences were observed between the two groups (I^2^ = 95.2%, p_Q_ < 0.001, p_subgroupdifference_ = 0.003), with the first group (European region, 7 studies) presenting a pooled prevalence of 2.5% (95% CI: [0.0170, 0.0385]), while the second group (Non-European region, 19 studies) reported a prevalence of 6.9% (95%CI: [0.0415, 0.1150] (Figure 3). Furthermore, meta-regression analyses were performed to explore the role of age, EDSS, and disease duration in the prevalence of DΜΙΙ among PwMS.

Age did not appear to have any effect on the prediction of DMII prevalence among PwMS (I^2^ = 95.8%, pQm = 0.30) (SF1). Differences in the presentation of EDSS score (mean values and median values) among the included studies lead to the performance of two different meta-regression analyses by stratifying the studies respectively, after the exclusion of 6 studies [15,16,23,24,29] that either did not provide data on EDSS score or used a different score (e.g., PDSS). Median EDSS and Mean EDSS also had no effect on the prediction of DΜΙΙ prevalence among PwMS (I^2^ = 77.5%, pQm = 0.1949 and I^2^ = 89.7%, pQm = 0.539, respectively) (SF2). Lastly, a meta-regression analysis based on disease duration was performed after the exclusion of three studies [17,32,33] that did not provide the mean disease duration. No statistically significant results were reported on the effect of disease duration on the prevalence of DΜΙΙ among PwMS (I^2^ = 96.82, pQm = 0.838) (SF3).

**Figure 4:**
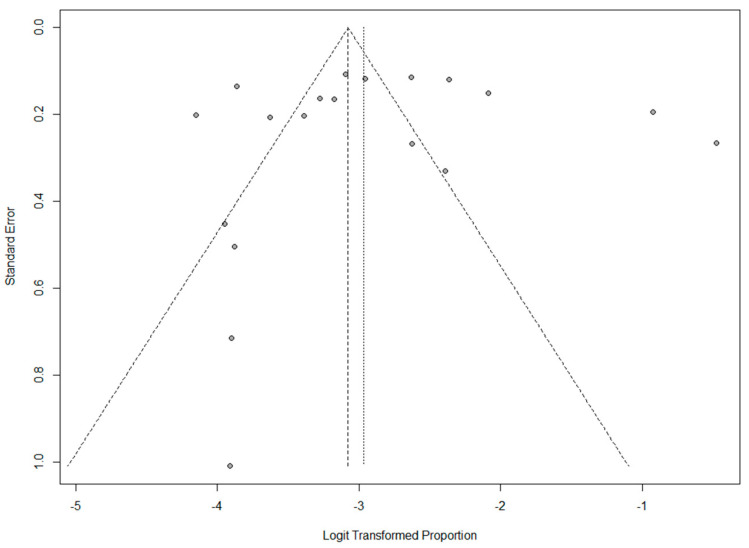
Funnel plot.

Publication Bias was assessed by funnel plot asymmetry and Egger’s linear regression test. In the case of the pooled proportions of DMΙΙ among PwMS, there was low funnel plot asymmetry (Figure 4). Although the funnel plot demonstrated asymmetry; publication bias was not confirmed by Egger’s test (*p* = 0.896).

A total of 19 studies with 15,360 PwMS were included in the meta-analysis. The mean age of the participants in the included studies was 46 years and the gender ratio was 71.18% female participants. Based on the results of the main meta-analytical process, utilizing the random effect model, we found a 5% prevalence of DΜΙΙ in the MS population.

Secondary analyses, using meta-regression, revealed that MS-specific characteristics, specifically EDSS (*p* = 0.19, *p* = 0.530) and disease duration (*p* = 0.89), as well as demographic characteristics, mainly age (*p* = 0.30), did not affect the prevalence of DΜΙΙ in PwMS. Additionally, a subgroup analysis based on region, with the studies being stratified into two separate groups (European and Non-European) revealed a statistically significant difference in the prevalence of DΜΙΙ in PwMS. The prevalence of DΜΙΙ was 2.5% for studies conducted within Europe [17,21,24,31,32,33], while for non-European studies the DM-II prevalence was 6.9%. 

The prevalence of DΜΙΙ in PwMS was lower than in the general population (9.6–10.3%) [34], and the results of the subgroup analysis were consistent with previous data regarding the prevalence of DΜΙΙ in different regions. A plethora of factors may account for this variance, such as sedentary behaviors, obesity, and insufficient physical activity, which have been linked to the development of DΜΙΙ and vary highly between Europeans and Americans [35,36,37]. Americans tend to spend 55% of their waking time engaged in sedentary behaviors in contrast to 40% for the European population [38] and have a 36.5% obesity prevalence compared to 15.9% [39].

The presence of cardiovascular comorbidities in PwMS seems to be related to worse clinical outcomes, higher admission risk, accelerated disease and disability progression, higher economic and psychological burden, and increased mortality rates. Additionally, PwMS and high comorbidity burden (at least three comorbid conditions) appear to have a higher two-year relapse rate [22,23].

Regarding the negative interactions between DMII and MS, in a recent study, Zivadinov and colleagues showed that the presence of DΜΙΙ in PwMS was accompanied by more severe and unconventional MRI outcomes, as well as higher rates of whole-brain, cortical, and gray matter volumes decrease (*p* < 0.05) [40]. Furthermore, Maric and colleagues found that the presence of DMII seems to be more frequent in progressive PwMS, which is attributed to the average age difference between progressive and relapse PwMS. Overall, the presence of DMII seems to accelerate the disease progression and disability status of PwMS and is associated with lower walking speed and a quicker decline of fine upper motor skills [2,40]. It is worth mentioning that pain is another element affected by the comorbid relationship and interaction of MS and DMII. In the case of MS, pain is a common symptom with high prevalence, known as chronic neuropathic pain, and is usually associated with lesions in the periventricular white matter, the lateral medial thalamic regions, and the spinal cord. According to Fiest and colleagues, PwMS with comorbid diabetes had increased pain accumulation rates during the second year of observation (risk ratio 2.42, CI:1.25–4.68) [18]. 

Etiologically, there is evidence of some pathophysiological and pharmacological underlying MS mechanisms that suggest an increased risk of DMII development in PwMS. Insulin resistance, oxidative stress, and adiposity seem to be potentially candidate pathophysiological mechanisms in PwMS. Beside the MS-specific effects of insulin resistance, which is associated with increased disease aggravation, higher EDSS score, and acceleration of disability [41], comorbid DMII has been negatively linked with pancreatic isle cell function, resulting in higher blood glucose levels and ultimately the manifestation of prediabetes or DΜΙΙ. Wens and colleagues demonstrated that PwMS had a higher risk of presenting with impaired glucose tolerance, which may increase the risk of developing cardiovascular comorbidities, including DΜΙΙ [42]. In addition, insulin resistance is associated with higher BMI and lower physical activity levels, which are also risk-factors for the development of DMII [43]. In addition, glucocorticoid therapy, which is commonly used for the management of MS relapses due to its anti-inflammatory and immunosuppressive properties, has been linked to hyperosmolar hyperglycemic nonketotic syndrome as well as new onsets of DMΙΙ [44,45]. Furthermore, some disease modifying and non-modifying treatments (i.e., glatiramer acetate, interferon beta-one alpha) have been associated with increased cardiovascular disease risk and specifically with increased diastolic blood pressure and plasma glucose [46]. In the case of Interferon-beta one alpha, the elevation or neutralization of tumor necrosis factor-a may increase insulin sensitivity, which is often accompanied by cytotoxicity to pancreatic isle cells, as a result of elevated interleukin-1 serum levels [47]. 

Traditionally, autoimmune pathogeneses have been attributed to CD4(+) T lymphocytes, as in MS, rheumatoid arthritis, and DMII, and to B lymphocytes, as in myasthenia gravis and systemic lupus erythematosus. This is because their primary genetic associations are mostly with certain human leukocyte antigen class II alleles, whose gene products present antigens to CD4(+) T cells. Because few autoimmune diseases show stronger associations with major histocompatibility complex class I alleles (ankylosing spondylitis, Bechet’s disease, and psoriasis), CD8(+) T cells, which interact with major histocompatibility complex class I molecules, have also been considered as key players in autoimmunity. A variety of pathological findings have highlighted the key role of CD8(+) cell populations, particularly in MS. First, CD8(+) cell associations with major histocompatibility complex class I alleles. Second, the predominance of CD8(+) T cells in demyelinating lesions. Furthermore, several clinical trials of monoclonal antibodies specifically against CD4(+) T cells, or the polarizing cytokines on which they depend, have failed to show any therapeutic benefit in MS, unlike broader-spectrum antibodies that deplete all T cells. In addition, a key element in the development of clinical metabolic syndrome and/or DMII is adipose tissue inflammation, which involves the activation of CD8(+) T cells. In contrast to people with low to normal BMI, in obese individuals there is an increase in CD8(+) T cells, which may lead to increased levels of IFN-γ [48,49,50,51]. 

Additionally, lifestyle factors such as excessive weight gain, smoking, and sleep disturbances as well as low physical activity levels either due to disability or as a means to conserve energy and prevent fatigue manifestations in PwMS, may progress to what is known as “exercise deficiency phenotype”, which is characterized by (a) increased accumulation of abdominal fat, (b) higher triglycerides levels, (c) decreased HDL levels, and (d) insulin resistance [52].

Finally, a recent study by Marie and colleagues found that the clinical phenotype of MS is not associated with worse blood pressure or glucose control outcomes. In addition, the authors found that the value of HbA1c in PwMS was lower than 7% [53]. 

## 4. Preventive and Management Strategies 

### 4.1. Multiple Sclerosis

There are no preventive strategies regarding the occurrence of MS. Regular clinical, radiological, and biomarker follow-up seems, up to today, to be the safest means of holistically following up such a multi-faceted disease [54].

### 4.2. Diabetes Mellitus Type II

Based on World Health Organization guidelines, specific preventative measures are necessary regarding the clinical manifestation of DMII. These include the preservation of a healthy BMI as per guidelines, a minimum of 30 min per day physical exercise of any kind, where it is advised to alternate between the various exercise categories, up to a moderate heart rate limit, a maintenance of a healthy diet profile with minimal sugar and saturated fat levels, and finally a complete and permanent smoking cessation of tobacco or other means, which are equally harmful [55].

### 4.3. Vitamin D

In people with a genetic or hereditary disposition for either MS or DMII, increasing serum Vitamin D levels (either by increasing sun exposure, or via administration of oral supplements) may decrease the odds of developing either condition. In the case of MS, increased vitamin D levels before the age of 20 was associated with a decreased risk of developing MS later in life, while higher Vitamin D levels correlated with decreased axonal damage in PwMS. Furthermore, Vitamin D deficiency has been linked with insulin resistance and increased odds of developing DMII [54,56].

### 4.4. Lifestyle Factors

Obesity has been linked with increased occurrence of DMII and more severe DMII symptomatology, as well as lower functionality levels and higher disability levels in PwMS. A healthy, balanced diet significantly lowers the occurrence of cardiovascular conditions, including DMII. Additionally, regular aerobic and weight exercise is linked to DMII prevention and efficient DMII management, as well as with increased functionality and slower disability rate progression in PwMS [54,55,56,57].

## 5. Strength and Limitations

To the best of our knowledge, this is the first attempt to calculate the prevalence of diabetes type II in the MS population. The main limitation of this study is the high heterogeneity between the included studies (I^2^ = 95%). Additionally, the authors could not draw conclusions about the cause-and-effect direction of the two investigated conditions because no data regarding the onset of DΜΙΙ (pre or post MS diagnosis) were provided. Lastly, missing information, such as EDSS scale scores and MS or DMII disease duration, as well as different types of data presentation (mean or median), interfered with the meta-analytical process. Given the interactions of the two conditions, the authors would suggest that physicians ought to update their clinical practices when treating PwMS with pre-diabetes or with DΜΙΙ.

## 6. Conclusions

Even though the prevalence of DMII in the MS population is lower than 10%, the interactions between the two conditions create significant challenges for patients, caregivers, and physicians. The presence of DMII in PwMS follows the same region pattern with the general MS population, and it does not seem to be affecting its prevalence. Given the fact that DΜΙΙ has a higher prevalence in males than in females and the fact that the majority of the included PwMS are females, the authors would hypothesize that MS may increase the risk of developing DMII. However, there is no available data to corroborate such a hypothesis. DΜΙΙ should be systematically recorded in MS, to clearly delineate any potential relationship. Additionally, there is a need for more studies structured around the interactions of MS and DMIΙ and assessing the direction of potential causation between those two conditions.

## Figures and Tables

**Table 1:** Included Studies.

Author	Year	Country	DMII Cases	N	Age	EDSS	Disease Duration	Female %	Comments
Hussein [15]	2006	Saudi Arabia	81	1206	-	-	9.9	64.1	
Kang [16]	2010	Taiwan	77	898	-	-		61	
Moccia [17]	2015	Naples	5	265	42.2	-	8.2		
Fiest [18]	2015	Canada	38	949	48.6	2.5	15.4	75.2	median EDSS
Pinhas-Hamiel [19]	2015	Israel	37	130	55.8	5.5	18.2	72.3	
Tettey [20]	2016	Australia	4	198	47.4	3	6	72	median EDSS
Sicras Mainar [21]	2017	Catalonia	15	222	45.5	3.2	13.4	64	
Kowalec [22]	2017	Canada	25	764	48.2	2.5	15.5	76.6	median EDSS
Conway [23]	2017	USA	90	2083	43	-	6.1	74.4	PDSS instead of EDSS
Murtonen [24]	2018	Finland	39	1074	-	-	-	70.6	
Flauzino [25]	2019	Brazil	10	119	42.8	3.2	43.1	68	
Chen [26]	2019	Australia	24	929	51.6	-	13	80.6	PDSS instead of EDSS
Ciampi [27]	2020	Chile	50	453	41	2	10.3	70.6	median EDSS
Maric [28]	2020	Serbia	56	2725	55.8	4	21.6	69.8	median EDSS
Pangan Lo [29]	2020	Australia	75	1518	55.7	-	20.5	79.6	
Fahmi [30]	2020	Egypt	23	60	31.4	2.8	4.3	68.3	
Pasic [31]	2021	Croatia	2	101	42.9	3.1	13.5	74.2	
Stanikic [32]	2022	Swiss	25	1615	47		11	73.3	median age & duration, SDRSS instead of EDSS
Silva [33]	2023	Portugal	1	51	38.2	1	3	66.7	median disease duration & EDSS

Note. EDSS: Expanded disability severity scale, PDSS: Patient determined disease steps, SDRSS: Self-reported Disability Status Scale; in the cases of no comment, the continuous measures are reported as means.

## Data Availability

Data are available upon reasonable request from the corresponding author.

## Supplementary Materials

The following supporting information can be downloaded at: https://www.mdpi.com/article/10.3390/jcm12154948/s1, Table S1: Observational study assessment using the PLOS ONE checklist [9]; Table S2: JBI Cross-Sectional Study Assessment [10].
