# Supplementary material for: The Prevalence of Diabetes Mellitus Type II (DMII) in the Multiple Sclerosis Population: A Systematic Review and Meta-Analysis
Source: J Clin Med. 2023 Jul 27;12(15):4948. doi: 10.3390/jcm12154948 (PMC10420178; doi:10.3390/jcm12154948)
Supplement: Supplementary file 1 [file jcm-12-04948-s001.zip › jcm-2478980-supplementary.pdf]

Supplementary materials

Search Algorithm

[Multiple sclerosis] AND [Diabetes mellitus type II] , Multiple Sclerosis AND [metabolic syndrome] , “Multiple sclerosis” AND “Diabetes mellitus type II” , “Multiple sclerosis” AND “ metabolic syndrome”

Population: Multiple Sclerosis, I: not applicable, C: not applicable , O: Diabetes mellitus type II

Table S1: Observational study assessment using the PLOS ONE checklist [1]

	Hussein et al 2006	Fiest et al 2015	Kowalec et al 2017	Conway et al 2017	Murtonen et al 2018	Flauzino et al 2019	Chen et al 2019	Ciampi et al 2020	Maric et al 2020	Pangan Lo et al 2020	Stanikic et al 2022	Silva et al 2023
Have the authors proposed a clear research question? Does the discussion of related literature in the Introduction section justify the research question? Have the authors cited and discussed other relevant literature on this topic?	Y	Y	Y	Y	Y	Y	Y	Y	Y	Y	Y	Y
Are the methods explained in detail, and are they appropriate for the study design? Are the statistical analyses appropriate?	Y	Y	Y	Y	Y	Y	Y	Y	Y	Y	Y	Y
Do the reported conclusions address the research question?	Y	Y	Y	Y	Y	Y	Y	Y	Y	Y	Y	Y
Have sample size calculations been provided? Are they appropriate given the sampling methodology?	Y	Y	Y	Y	Y	Y	Y	Y	Y	Y	Y	Y
Have covariates and confounding variables been reported and defined in the Methods section?	N	Y	Y	Y	N	Y	Y	Y	Y	N	Y	Y
Are the results presented in tables in the Results section uniform and mathematically correct?	N	Y	Y	Y	Y	Y	Y	Y	Y	Y	Y	Y
Are the study limitations on the effects of the study outcomes thoroughly discussed?	N	N	Y	Y	Y	N	Y	N	N	Y	Y	N

Table S2: JBI Cross-Sectional Study Assessment [2]

	Kang et al 2010	Moccia et al 2015	Pinhas-Hamiel et al 2015	Tettey et al 2016	Sicras-Mainar et al 2017	Fahmi et al 2020	Pasic et al 2021	Silva et al 2023
Were the criteria for inclusion in the sample clearly defined?	Y	Y	Y	Y	Y	Y	Y	Y
Were the study subjects and the setting described in detail?	N	N	Y	Y	Y	Y	Y	Y
Was the exposure measured in a valid and reliable way?	Y	Y	Y	Y	Y	Y	U	Y
Were objective, standard criteria used for measurement of the condition?	Y	Y	Y	Y	Y	Y	Y	Y
Were confounding factors identified?	N	N	Y	Y	Y	Y	Y	Y
Were strategies to deal with confounding factors stated?	Y	Y	Y	Y	Y	Y	Y	Y
Were the outcomes measured in a valid and reliable way	Y	Y	Y	Y	Y	Y	Y	Y
Was appropriate statistical analysis used?	Y	Y	Y	Y	Y	Y	Y	Y

Figure S1: Age-Dm2 cases Bubble plot

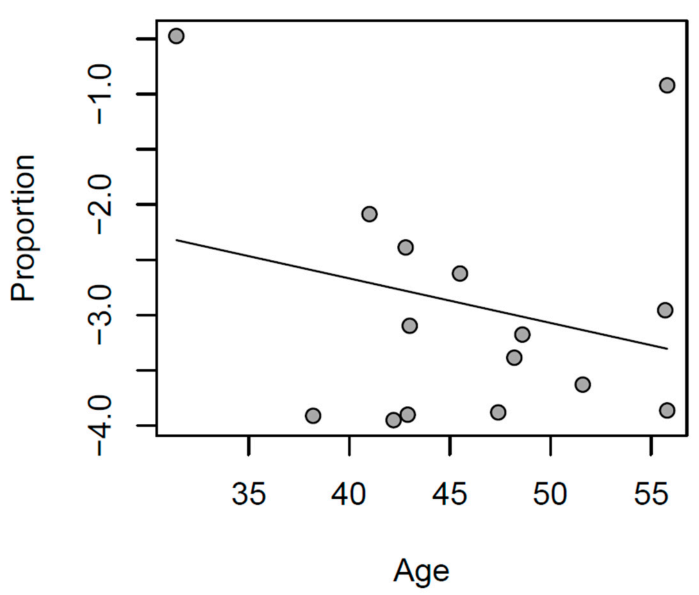

Figure S2 : Median EDSS – Dm2 cases and Mean EDSS-Dm2 cases bubble plots

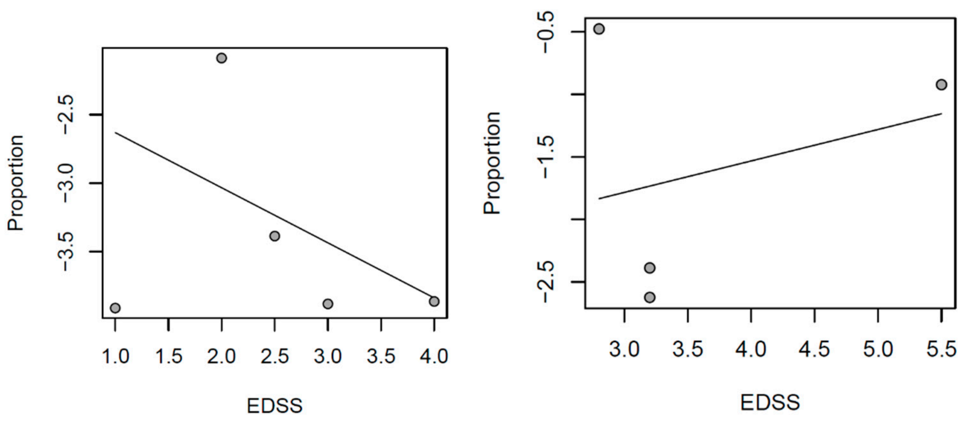

Figure S3: Disease duration-Dm2 cases bubble plot

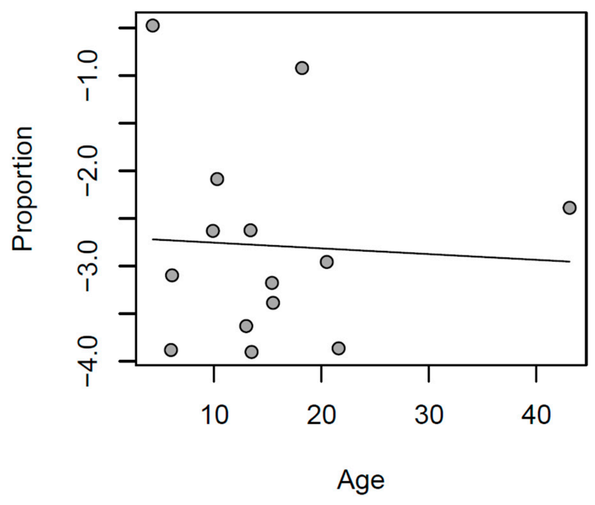

Supplementary materials references

1. Downes MJ, Brennan ML, Williams HC, et al. Development of a critical appraisal tool to assess the quality of cross-sectional studies (AXIS). BMJ Open 2016;6:e011458. doi: 10.1136/bmjopen-2016-011458.

2. Moola S, Munn Z, Tufanaru C, Aromataris E, Sears K, Sfetcu R, Currie M, Qureshi R, Mattis P, Lisy K, Mu P-F. Chapter 7: Systematic reviews of etiology and risk . In: Aromataris E, Munn Z (Editors). Joanna Briggs Institute Reviewer's Manual. The Joanna Briggs Institute, 2017
